# Supplementary figures and images for: Healthcare professionals interpersonal variability and determinants of medical decision thresholds for active management of extremely preterm infants in a level 3 perinatal center in France
Source: PLoS One. 2025 Apr 3;20(4):e0320900. doi: 10.1371/journal.pone.0320900 (PMC11967952; doi:10.1371/journal.pone.0320900)

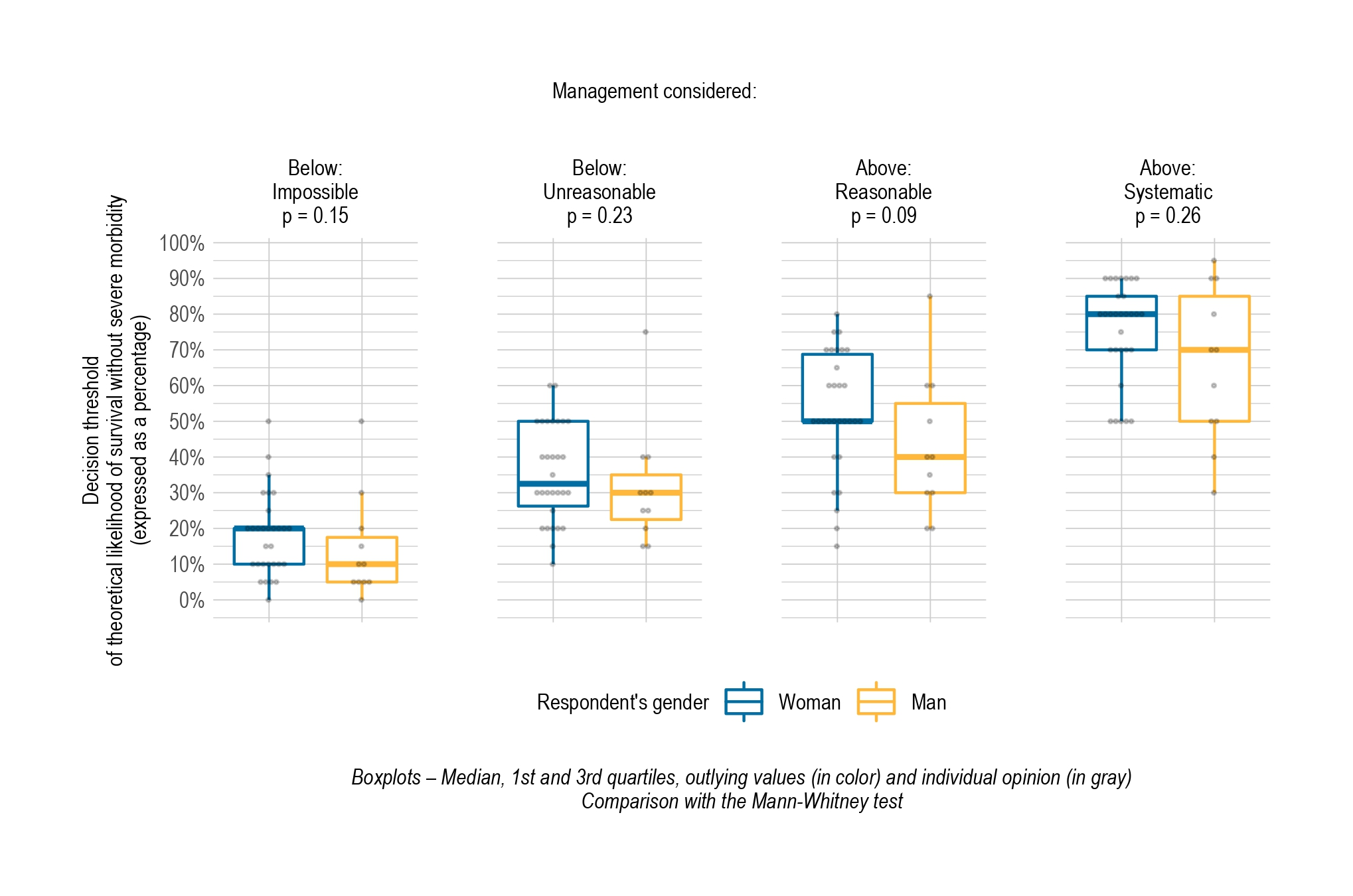

Supplement: S1 Fig — For active management for each of the four attitudes in the EXPRIM protocol. (TIF) [file pone.0320900.s001.tif]

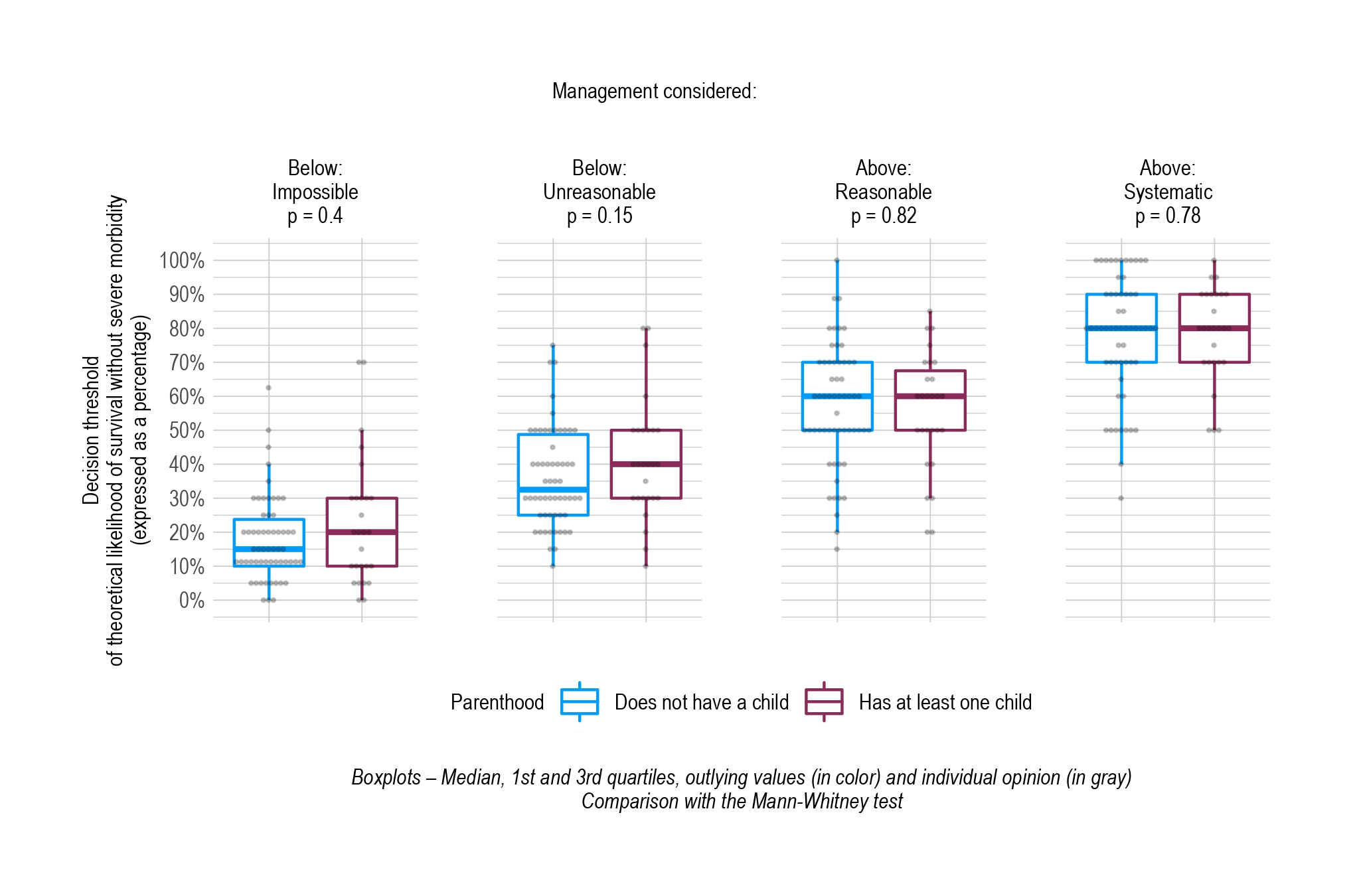

Supplement: S2 Fig — For active management for each of the four attitudes in the EXPRIM protocol. (TIF) [file pone.0320900.s002.tif]

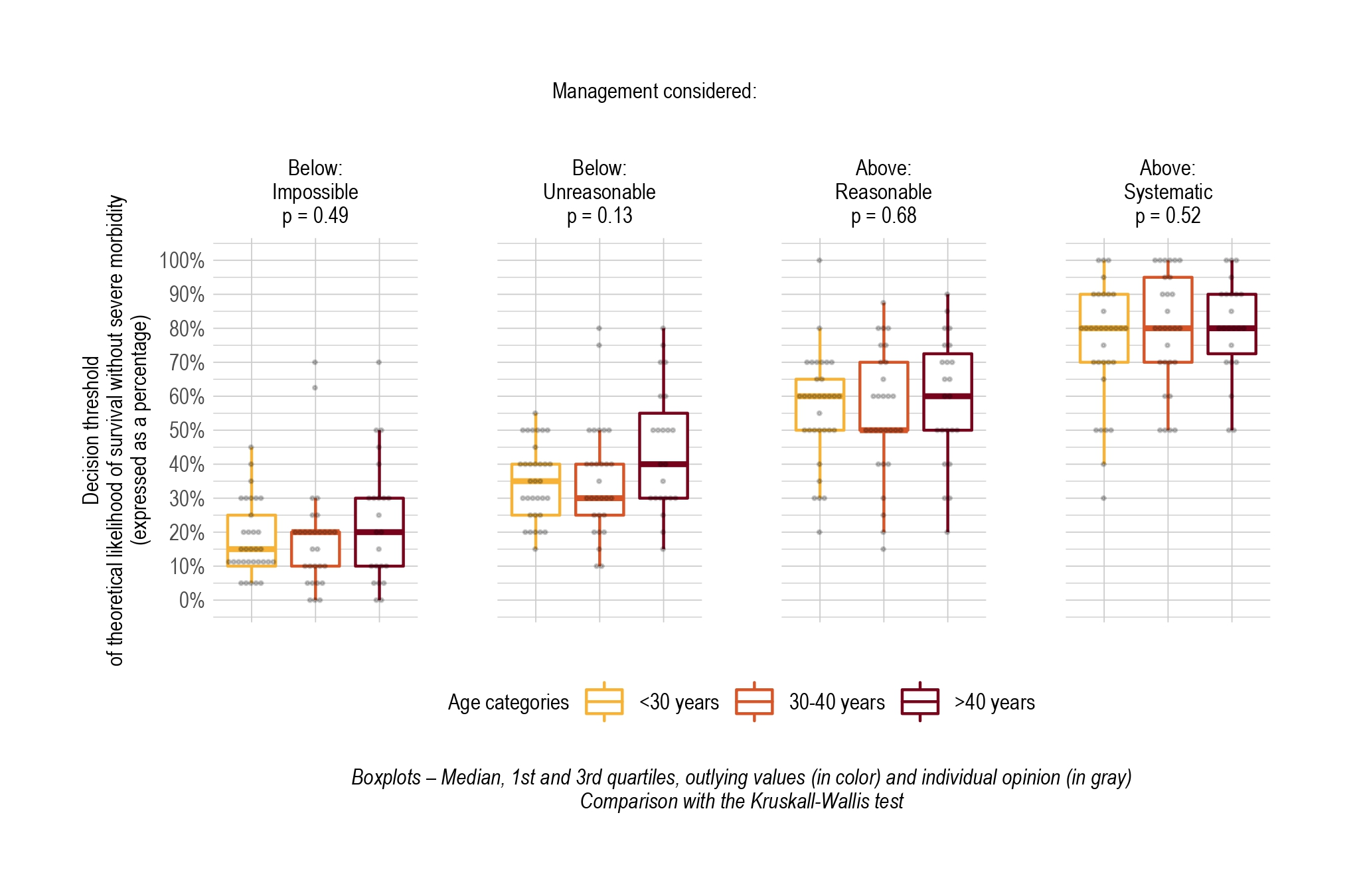

Supplement: S3 Fig — For active management for each of the four attitudes in the EXPRIM protocol. (TIF) [file pone.0320900.s003.tif]

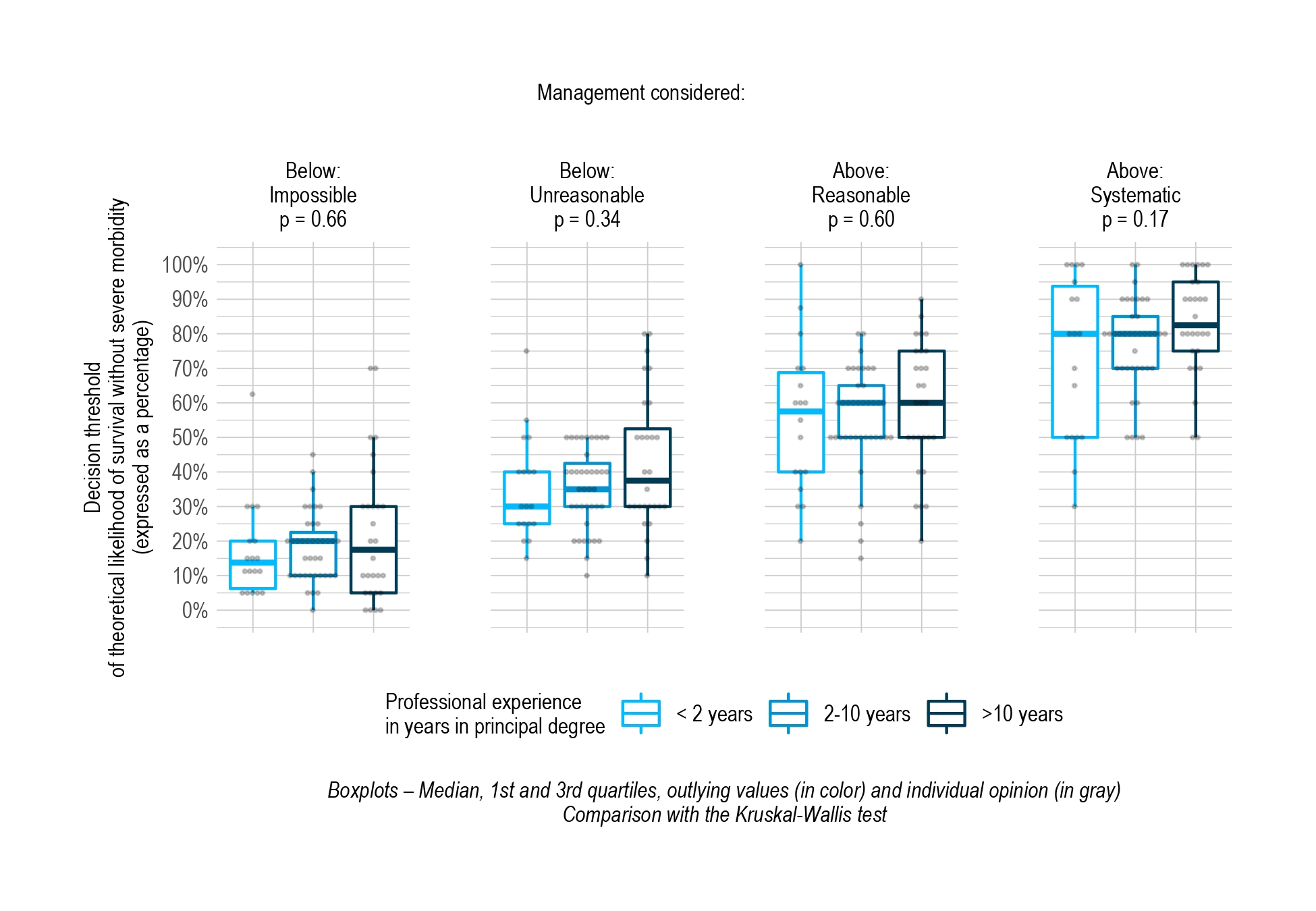

Supplement: S4 Fig — For active management for each of the four attitudes in the EXPRIM protocol. (TIF) [file pone.0320900.s004.tif]

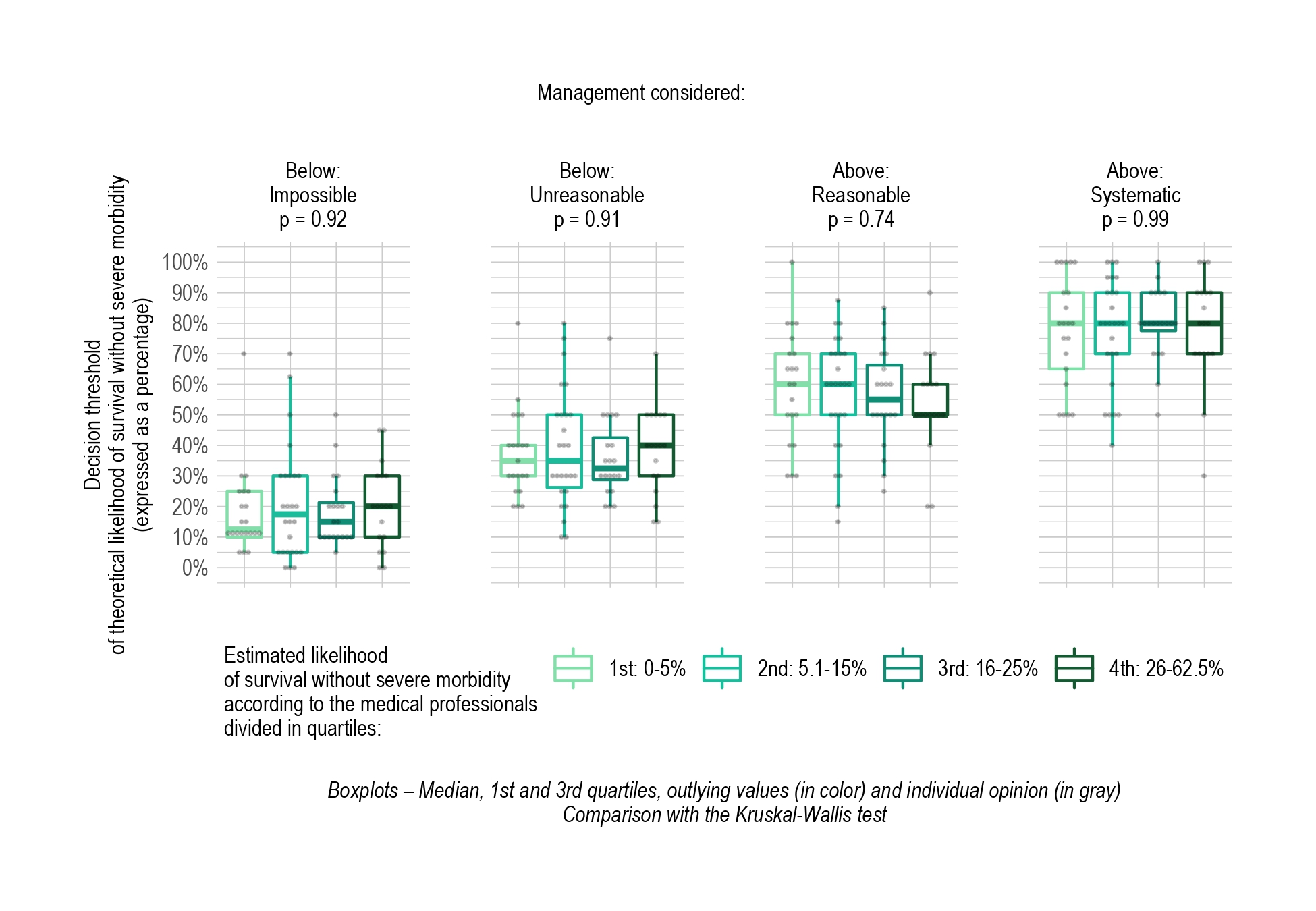

Supplement: S5 Fig — For active management for each of the four attitudes in the EXPRIM protocol. (TIF) [file pone.0320900.s005.tif]

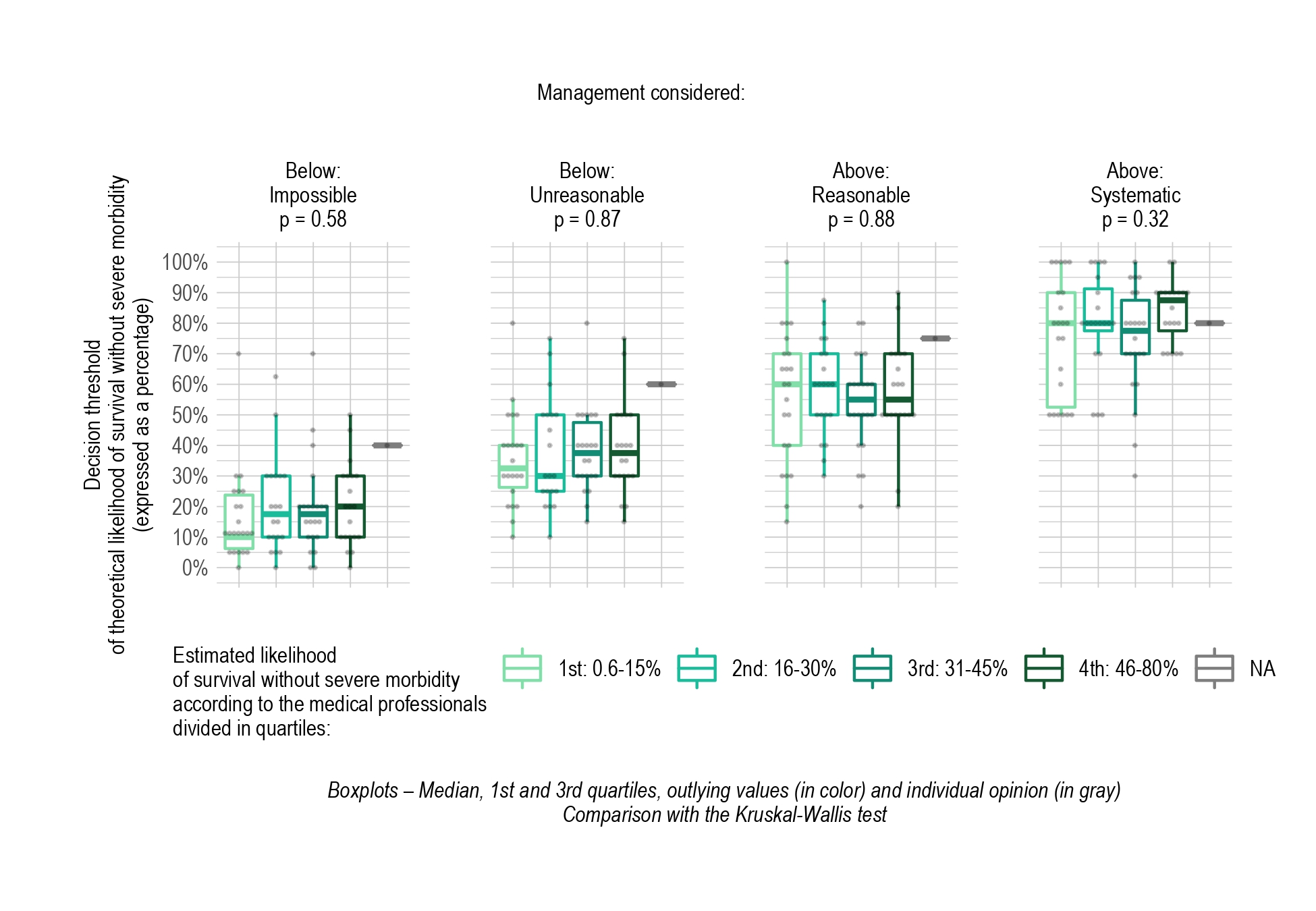

Supplement: S6 Fig — For active management for each of the four attitudes in the EXPRIM protocol. (TIF) [file pone.0320900.s006.tif]

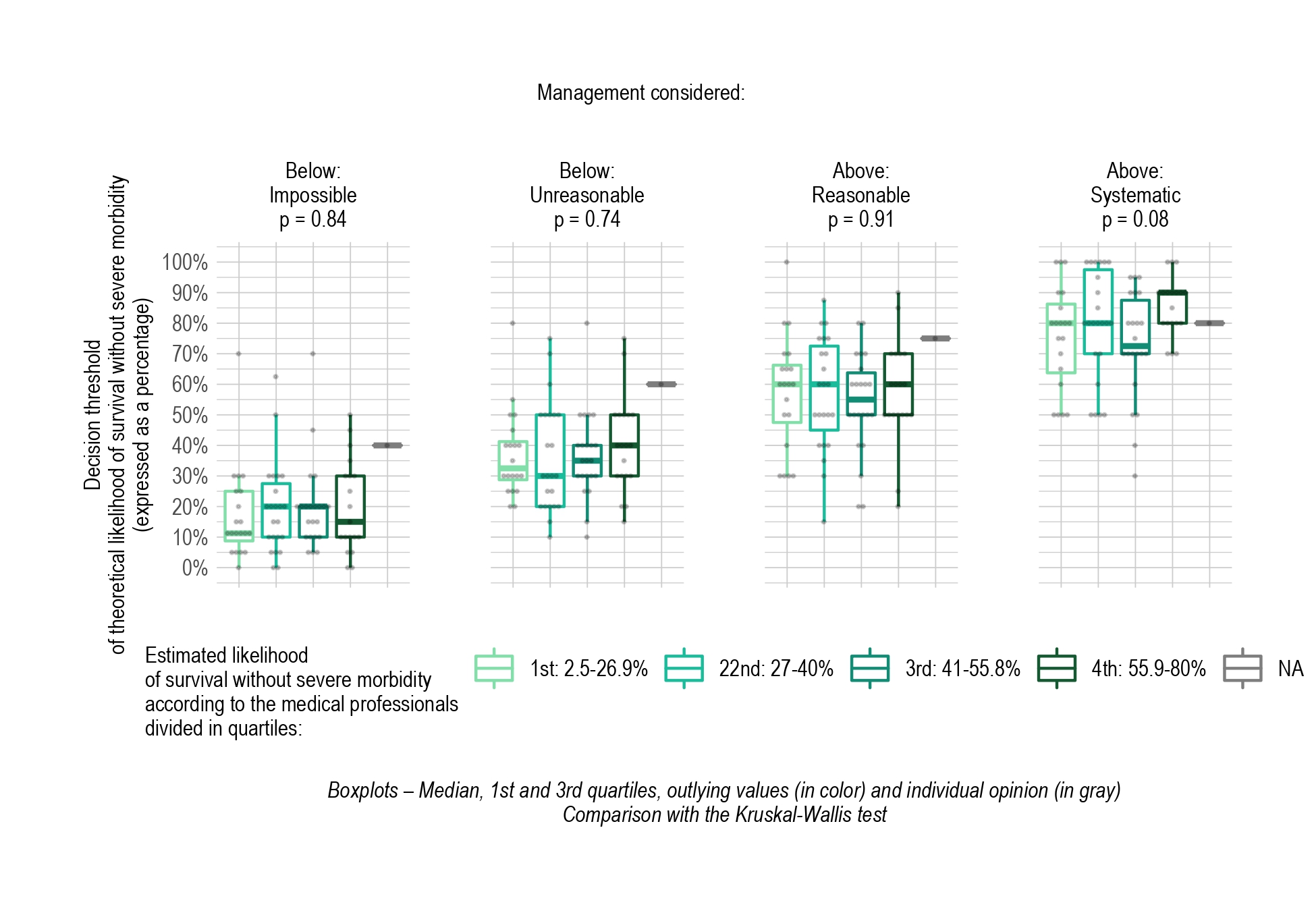

Supplement: S7 Fig — For active management for each of the four attitudes in the EXPRIM protocol. (TIF) [file pone.0320900.s007.tif]

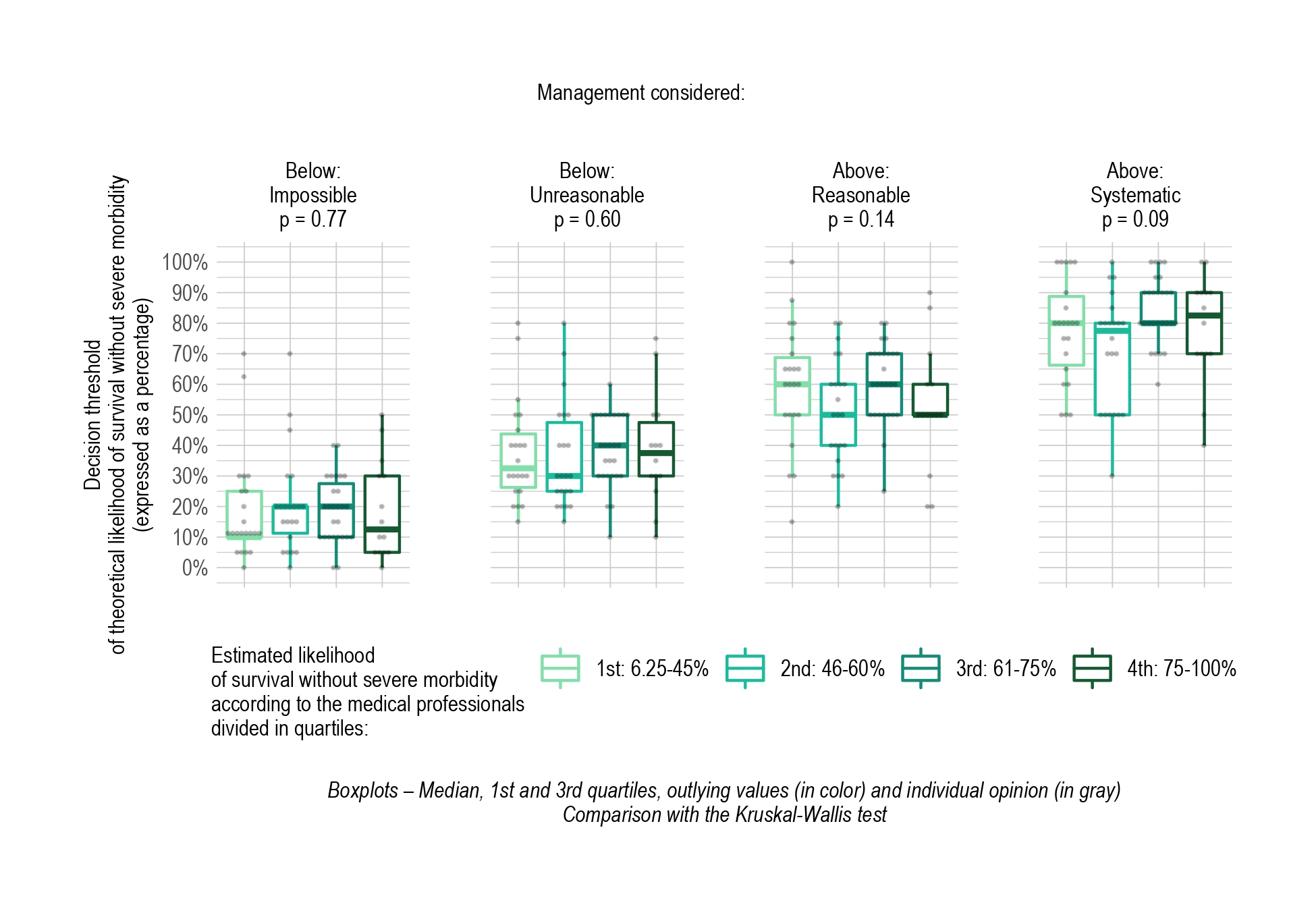

Supplement: S8 Fig — For active management for each of the four attitudes in the EXPRIM protocol. (TIF) [file pone.0320900.s008.tif]
