# Supplementary material for: Healthcare professionals interpersonal variability and determinants of medical decision thresholds for active management of extremely preterm infants in a level 3 perinatal center in France
Source: PLoS One. 2025 Apr 3;20(4):e0320900. doi: 10.1371/journal.pone.0320900 (PMC11967952; doi:10.1371/journal.pone.0320900)
Supplement: S9 Appendix — (DOCX) [file pone.0320900.s009.docx]

Appendix 1: Questionnaire

**PAGE 1/2**

**Active management of extremely preterm infants at 23 and 24 weeks:
Is it reasonable?**

**Introduction: Who are you?** – This questionnaire is anonymous: Check the boxes corresponding to your situation

| Obstetric team |  |  | Woman |  |
| --- | --- | --- | --- | --- |
| Pediatric team |  |  | Man |  |
|  |  |  |  |  |
| Resident |  |  | Year of birth |  |
| Staff physician |  |  |  |  |
| Midwife |  |  | Years since diploma |  |
| Childcare assistant |  |  |  |  |
| Infant nurse, state certification |  |  | Do you have any children? |  |
| Nurse, state-certification |  |  |  |  |

**DEFINITIONS: To understand completely** – Please read the following 3 definitions.

**1). Study population:** Children born between 23^0/6^ and 24^6/7^ weeks, and:

- Inborn birth in a level-3 hospital
- Viable: estimated birth weight before birth > 500 g
- Complete corticosteroid treatment
- Complete loading dose of magnesium sulfate (30 min)
- Active resuscitation

**2). Morbidity**: estimated at discharge from the neonatology unit, at least one of the following:

|  | **Severe morbidity (considered here)** | **Very severe morbidity (for information)** |
| --- | --- | --- |
| **Cerebral white matter lesions** | Cystic periventricular leukomalacia, unilateral or bilateral | Cystic periventricular leukomalacia, bilateral |
| **Cerebral hemorrhagic lesions** | IVH grade 3 (associated with ventricular dilation) and 4 (associated with hemorrhagic damage to adjacent parenchyma). | IVH grade 4 |
| **Ocular damage** | Preterm retinopathy grade 3 and/or requiring laser management | Preterm retinopathy, with laser management |
| **Gastrointestinal involvement** | Severe necrotizing enterocolitis (NEC): that is, type 2 or 3 of Bell's classification | NEC requiring surgical management |
| **Pulmonary involvement** | Moderate or severe bronchopulmonary dysplasia: that is, requiring oxygen therapy at 36 weeks | Severe bronchopulmonary dysplasia: that is, requiring oxygen therapy >30% and/or mechanical ventilatory support (endotracheal or noninvasive) at 36 weeks |

**PAGE 2/2**

**3). Classification of the obstetric-pediatric decision about active or palliative care for pregnancies at risk of extremely preterm birth**

| **Situation** | **Obstetric-pediatric decision about management at birth** | **Involvement of parents in the management decision** |
| --- | --- | --- |
| **Impossible** | Systematic palliative care | Parents cannot demand care deemed excessive |
| **Unreasonable** | Active treatment inadvisable, but acceptable if parents want it | The parents' request should be respected |
| **Reasonable** | Active management advised but parental request for palliative care acceptable | The parents' request should be respected |
| **Systematic** | Systematic active management | Parents cannot refuse this care, in the best interests of the child |

**Part 1: Your opinion about the acceptability of active management in the population studied** – Put a cross on the scale at the threshold (expressed as a percentage) that you consider appropriate.

| In your opinion, what is the threshold for the likelihood of survival without severe morbidity below which active management seems impossible?  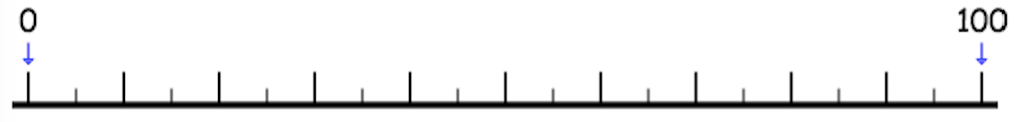 |
| --- |
| In your opinion, what is the threshold for the likelihood of survival without severe morbidity below which active management seems unreasonable?  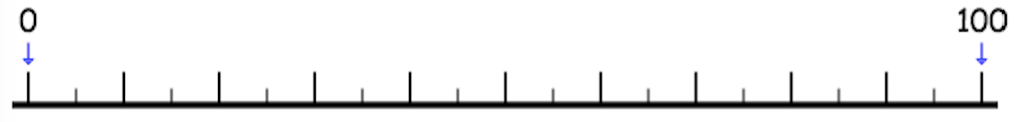 |
| In your opinion, what is the threshold for the likelihood of survival without severe morbidity above which active management seems reasonable?  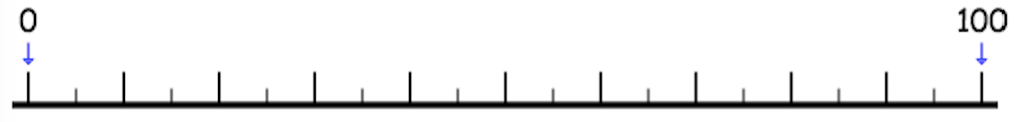 |
| In your opinion, what is the threshold for the likelihood of survival without severe morbidity below which active management should be systematic?  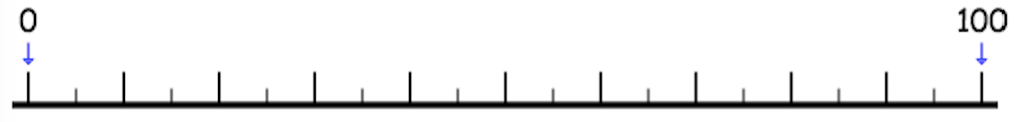 |

**PART 2: Your knowledge of the outcome of the population studied -** (+ that of the children born at 25^0/7^—25^6/7^weeks)

To your knowledge..., what is the percentage of survival without severe morbidity in each category?

| **Sex, birth weight and age at birth** | Boy  500-600 g | Girl  500-600 g | Boy  600-700 g | Girl  600-700 g | Boy  > 600-700 g | Girl  > 600-700 g |
| --- | --- | --- | --- | --- | --- | --- |
| 23^0/7^ —23^3/7^ weeks |  |  |  |  |  |  |
| 23^4/7^—23^6/7^ weeks |  |  |  |  |  |  |
| 24^0/7^—24^3/7^ weeks |  |  |  |  |  |  |
| 24^4/7^—24^6/7^ weeks |  |  |  |  |  |  |
| 25^0/7^— 25^0/7^ weeks |  |  |  |  |  |  |
