# Supplementary material for: Healthcare professionals interpersonal variability and determinants of medical decision thresholds for active management of extremely preterm infants in a level 3 perinatal center in France
Source: PLoS One. 2025 Apr 3;20(4):e0320900. doi: 10.1371/journal.pone.0320900 (PMC11967952; doi:10.1371/journal.pone.0320900)
Supplement: Attachment — Submitted filename: Reviewer for Manuscript Number PONE-D-24-19296.docx [file pone.0320900.s010.docx]

Esteemed;

Thank you. I found your paper work good learning opportunity on a sensitive and practical issue having significant differences in consideration despite similarity of developmental level amongst nations, including those in Europe.

Based on the criteria requisites for publication on PLOS ONE (1-7), I found it suitable for publication with minor revision, or explanations:

1. Data collection had finalized in April 2020, and the time lapsed towards publication concerns me; four years lapsed. I am worried this NICU level 3 might have changed its protocol/ approach by now.
2. I prefer methods only as a topic than methods and materials. There was no any material experimentation.
3. Table 1 contains limited data (can leave this comment if only mine amongst reviewers).
4. Results: table 1 years of work experience - we use 3 years as least experienced; is two years a cut point per French MOH? Some papers use five (5) years.
