# Supplementary material for: Healthcare professionals interpersonal variability and determinants of medical decision thresholds for active management of extremely preterm infants in a level 3 perinatal center in France
Source: PLoS One. 2025 Apr 3;20(4):e0320900. doi: 10.1371/journal.pone.0320900 (PMC11967952; doi:10.1371/journal.pone.0320900)
Supplement: Attachment — Submitted filename: Review_Hale.docx [file pone.0320900.s012.docx]

Variability and determinants of medical decisions thresholds for active management of extremely preterm infants in a level 3 hospital in France

Dear Editor

Thank you for allowing me to review this important article.

Dear Authors,

Thank you for the important work. I have enjoyed reading your article. However, I have some comments that can improve the readability of your manuscript.

Style:

I understand that this article might have been submitted to another journal. Please revise the format according to the PLoS one authors guideline. There are some sections which does not conform to the style of PLoS one's style. For example, key words should come immediately after conclusion, no need of article summary, what is known about this subject, what this study adds etc. Please revisit your style of manuscript writing.

Title: The title is confusing as it stands now. Please improve it. Propose titles: "Healthcare Professionals Interpersonal Variability in Medical Decision Thresholds for Active Management of Extremely Preterm Neonates in X Hospital, a level 3 healthcare institution in France."

Abstract:

The results section of the abstract is confusing. If readers decide to read just your abstract "The median threshold (threshold of what?) below which active management was considered not acceptable was 15% (IQR 10-30%). The median threshold (threshold of what?) above which active management could not be refused was 80% (IQR 70- 90%). It varied significantly by profession (p=0.005)." does not give meaning. I understand what you meant after reading the whole manuscript but reading just the abstract, does not give any meaning. Please revise it.

In general, your abstract does not reflect the main findings of you manuscript.

Key words:

Your title must be formed by your key words. In your article, the keywords are not reflected in your title. Please revise your keywords accordingly.

Methods:

In your methods section, the way you classifed age groups and levels of experience sounds random. What is the basis for classifying age groups and level of exprience for your study? Please define the years for junior, mid level, and senior based on evidence and then classify.

Results

In the methods section you stated that expereince was categorized as <2, 2-4, and >4 years but in Table 1 it is different. Pls be consistent.

Line 234" Five respondents considered that it was never impossible and set their threshold at 0%." Please rewrite this. I understand it but instead of using double negation, use a simple statement.

Discussion:

Please use the first paragraph to restate the aim, and summarize your findings including the numerical values of the relevant findings that need to be discussed. In subsequent paragraphs, please discuss each finding you summarized in the first paragraph to compare and contrast, state the reason for similarities or differences. The paragraph’s are lengthy and lacks clarity. Your discussion section needs a major revision.
